# Electro-Nano Diagnostic Platform Based on Antibody–Antigen Interaction: An Electrochemical Immunosensor for Influenza A Virus Detection

**DOI:** 10.3390/bios13020176

**Published:** 2023-01-23

**Authors:** Yudum Tepeli Büyüksünetçi, Ülkü Anık

**Affiliations:** 1 Sensors, Biosensors and Nano-Diagnostic Laboratory, Research Laboratory Center, Mugla Sitki Kocman University, Kotekli, 48000 Mugla, Turkey; 2 Chemistry Department, Faculty of Science, Mugla Sitki Kocman University, Kotekli, 48000 Mugla, Turkey

**Keywords:** influenza A virus, electrochemical immunosensor, multi-walled carbon nanotube gold-platinum hybrid nanomaterial, H1N1 virus

## Abstract

H1N1 is a kind of influenza A virus that causes serious health issues throughout the world. Its symptoms are more serious than seasonal flu and can sometimes be lethal. For this reason, rapid, accurate, and effective diagnostic tests are needed. In this study, an electrochemical immunosensor for the sensitive, selective, and practical detection of the H1N1 virus was developed. The sensor platform included multi-walled carbon nanotube gold-platinum (MWCNT-Au-Pt) hybrid nanomaterial and anti-hemagglutinin (anti-H1) monoclonal antibody. For the construction of this biosensor, a gold screen-printed electrode (AuSPE) was used as a transducer. Firstly, AuSPE was modified with MWCNT-Au-Pt hybrid nanomaterial via drop casting. Anti-H1 antibody was immobilized onto the electrode surface after the modification process with cysteamine was applied. Then, the effect of the interaction time with cysteamine for surface modification was investigated. Following that, the experimental parameters, such as the amount of hybrid nanomaterial and the concentration of anti-H1 were optimized. Under the optimized conditions, the analytical characteristics of the developed electrochemical immunosensor were investigated for the H1N1 virus by using electrochemical impedance spectroscopy. As a result, a linear range was obtained between 2.5–25.0 µg/mL with a limit of the detection value of 3.54 µg/mL. The relative standard deviation value for 20 µg/mL of the H1N1 virus was also calculated and found as 0.45% (*n* = 3). In order to determine the selectivity of the developed anti-H1-based electrochemical influenza A immunosensor, the response of this system towards the H3N2 virus was investigated. The matrix effect was also investigated by using synthetic saliva supplemented with H1N1 virus.

## 1. Introduction

Concerns about the reemergence of an influenza pandemic have been increased since low rates of COVID-19 were observed. It has been reported that the rate of seasonal influenza has dropped since April 2020 based on the COVID-19 precautions such as quarantine, travel restrictions, the closures of companies and schools, the usage of masks, etc. [1,2,3,4,5]. However, this long-term decrease in influenza virus infections might reduce herd immunity which could result in massive influenza spread [6,7].

The influenza virus, which belongs to the *Orthomyxoviridae* family [8], is a respiratory virus. Based on the variations of its matrix protein and nucleoprotein, the virus is classified as influenza virus A, influenza virus B, and influenza virus C [9,10]. Considering morbidity and harm, influenza A is recognized as the most dangerous type [11,12,13,14]. On the virus surface, there are two enclosed glycoproteins, namely, hemagglutinin (H) and neuraminidase (N), which play very important roles in the infection process of the virus. Until now, 18 H and 11 N subtypes have been verified [14,15,16].

Furthermore, H1N1 (swine flu) is a type of influenza A virus that transmits almost everywhere around the world [17]. As the influenza virus has 18 H types together with 11 N types, the management of its spread becomes complicated [18,19]. The last pandemic that was caused by a novel H1N1 strain was in 2009, which emerged from 1918 H1N1 strain [20,21,22], which caused 20–40 million deaths between 1918–1919 [14,23,24,25]. Additionally, it has been reported that swine flu can show more serious symptoms, such as persistent high fever, sore throat, chest pain, vomiting, etc., compared to seasonal flu [22,26,27]. For these reasons, rapid, sensitive, and effective detection of H1N1 is necessary. There are conventional methods for the diagnosis of swine flu such as cell culture, enzyme-linked immunosorbent assay (ELISA), conventional reverse transcription PCR, and real-time PCR. However, these methods need long time, specialized personnel, and expensive instruments [22,28,29,30,31]. Besides these techniques, rapid tests were also developed and utilized for the same purpose. As these tests are practical and possess point of care (POC) nature, they can be accepted as advantageous over those standard techniques. However, these rapid tests lack sensitivity and accuracy [10,32,33,34]. For example, the sensitivity that was obtained with these rapid tests was reported as 40 to 80% and 40 to 60% for seasonal influenza and swine flu, respectively [10]. Therefore, sensitive, accurate, and practical systems for swine flu diagnosis are tremendously needed. To date, electrochemical immunosensors containing many different modifying materials have been developed for the detection of H1N1 [14,35]. Veerapandian et al. reported the development of an electrochemical sensor platform composed of methylene blue-electro-adsorbed graphene oxide nanostructures modified with monoclonal antibodies against the H proteins of H1N1 [36]. Moreover, Han et al. developed a microfluidic electrochemical immunosensor for the detection of H1N1 virus using ZnO nanorods [37]. Additionally, Mikula et al. presented a dipyrromethene-copper complex-modified electrochemical biosensor for the detection of anti-H antibodies against swine flu H1N1 [38]. Considering the progress in the electrochemical systems for H1N1 virus detection, it is a fact that nanomaterials are frequently used in these systems to improve their performances.

Carbon nanotubes (CNTs), especially multi-walled carbon nanotubes (MWCNT), are one of the most used nanomaterials for electrochemical biosensors due to their unique features, such as large surface area, high conductivity, non-toxicity, ease in functionalization, and rapid electrode kinetics [39,40]. Additionally, to enhance the biosensor’s performance, MWCNT can be used as an appropriate material for the incorporation of noble metal nanoparticles (NP). The combination of CNTs and metal NPs causes a synergistic effect in terms of electrical connection and sensitivity [41].

In light of these facts, in the present study, an anti-H1-based electrochemical immunosensor was developed. MWCNT-Au-Pt was introduced into the developed system in order to increase the sensitivity and accuracy. The specific interaction between anti-H1 and H1N1 was monitored using electrochemical impedance spectroscopy (EIS). 

## 2. Materials and Methods

### 2.1. Reagents

MWCNT, HAuCl_4_·3H_2_O, H_2_PtCl_6_·6H_2_O, trisodium citrate, NaNO_3_, HNO_3,_ HCl, cysteamine, bovine serum albumin (BSA), 1-ethyl-3-(3-dimethylaminopropyl) carbodiimide hydrochloride (EDC), N-hydroxysuccinimide (NHS), Fe(CN)_6_]^4−^/[Fe(CN)_6_]^3^, graphite powder, ethylene glycol (EG), NaHCO_3_, NaCl, and KCl were obtained from Sigma-Aldrich, St. Louis, Missouri, ABD. KH_2_PO_4_, NaOH, H_2_SO_4,_ KMnO_4_, and H_2_O_2_ were purchased from Merck. In addition, influenza A hemagglutinin H3 monoclonal antibody (anti-H3), inactive influenza A/New Caledonia/20/99 (H1N1), and inactive influenza A/Brisbane/10/07 (H3N2) virus were purchased from HyTest, Turku, Finland. Virus solutions of different concentrations were prepared in pH 7.4 phosphate buffer with saline. All aqueous solutions were prepared using pure water. All the reagents were of analytical grade and were used without further purification.

### 2.2. Instruments and Procedures

EIS measurements were performed with a FRA-modeled μ-AUTOLAB potentiostat–galvanostat device that was supported by NOVA Software(version 1.10, Nova Software Inc., China), in the presence of 10 mM [Fe(CN)_6_]^−3/−4^ in pH 7.4, 50 mM phosphate buffer. The EIS working frequency range was set between 0.1 Hz–10 kHz at a potential value of 0.10 V. Throughout the entire study, a commercial 220 AT model Methrohm branded screen-printed gold electrode (AuSPE) was used, which contained the gold working electrode (diameter = 4 mm), gold auxiliary electrode, and silver reference electrode on a single platform.

The morphological and structural characterizations of the MWCNT-Au-Pt hybrid nanomaterial were carried out with scanning electron microscopy (SEM) and energy dispersive X-ray (EDX) analysis via a ZEISS ULTRA PLUS.

### 2.3. Hybrid Nanomaterials Preparation

#### 2.3.1. MWCNT-Based Hybrid Nanomaterials Preparation

For this purpose, activated MWCNTs were used. First of all, in order to perform the acid activation procedure, the proper amount of MWCNT powder was sonicated in 40 mL of acid mixture including H_2_SO_4_/HNO_3_ (3:1 *v*/*v*) for 6 h. After that, the activated MWCNT powder was rinsed with pure water, centrifuged, and then dried in a vacuum oven at 50 °C for 14 h. Then, platinum nanoparticle (PtNP) and gold nanoparticle (AuNP) or only AuNP deposition procedure on MWCNT was started with the sonication of 20 mg activated MWCNT in the presence of 20 mL of the Au and Pt nanoparticle solutions (for MWCNT-Au-Pt) or only AuNp (for MWCNT-Au) solution, which were prepared according to previous studies [35,36]. After the sonication step for 4 h, the mixture was annealed at 150 °C for 4 h to increase functionality. 

#### 2.3.2. Graphene-Based Hybrid Nanomaterial Preparation

In order to prepare graphene-Au and graphene-Pt, the first step was the synthesis of graphene oxide (GO) by the Hummers–Offeman method [37]. For this purpose, by stirring at 25 °C for 24 h, graphite powder was dispersed in 23 mL of 98% H_2_SO_4_ solution for the separation of the graphite layers. After 100 mg of NaNO_3_ was put into the final solution and mixed for 30 min, 3 mg of KMnO_4_ was added drop-by-drop to the medium, which the temperature was controlled by an ice bath to oxidize the graphite layers. Then, 46 mL of ultra-pure water was added to that solution, after the solution was heated up to 40 °C. As the final step of the Hummers method, 140 mL of ultrapure water and 10 mL of 30% H_2_O_2_ solution were put into the solution, respectively to stop the oxidation reaction with KMnO_4_.

After the residues of the reaction were eliminated via centrifuge, water was removed from the obtained GO powder.

Moreover, the graphene-Au and graphene-Pt hybrid nanomaterials were synthesized according to the study of Xu et al. [38]. First, 10 mg GO powder was dispersed in 10 mL of pure water and sonicated for 1 h to obtain a stable GO colloid [10]. Next, 20 mL of EG solution (reducing reagent) and 0.5 mL of 0.01 M HAuCl_4_·3H_2_O or H_2_PtCl_6_·6H_2_O (metal source) were added into the GO colloidal solution and mixed for 30 min. Then, after 6 h of the stirring procedure at 100 °C, the graphene-Au and graphene-Pt hybrid nanomaterials were obtained. For the separation of obtained hybrid nanomaterials from an excess of EG, centrifugation and a subsequent washing procedure with pure water for five times were applied.

In the last step, the graphene-Au and graphene-Pt hybrid nanomaterials were dried in an oven at 60 °C for 12 h. 

Before using electrode modification, all types of hybrid nanopowders were dispersed in pure water at a concentration of 10 mg mL^−1^ [10].

### 2.4. Preparation of Hybrid Nanomaterial Modified AuSPE

MWCNT-Au, MWCNT-Au-Pt, graphene-Au, and graphene-Pt hybrid nanomaterials were used separately to modify AuSPE. For this purpose, 10 µL of hybrid nanomaterial suspension (10 mg/mL in pure water) was dropped onto the working electrode surface and left until the suspension dehydrated at 25 °C for 15 min. At the end of this period, AuSPE/Grafen-Au, AuSPE/MWCNT-Au, AuSPE/Grafen-Pt, and AuSPE/MWCNT-Au-Pt nanomaterials were obtained. 

### 2.5. Fabrication of Anti-H1 Based Electrochemical Immunosensor

**Scheme 1:**
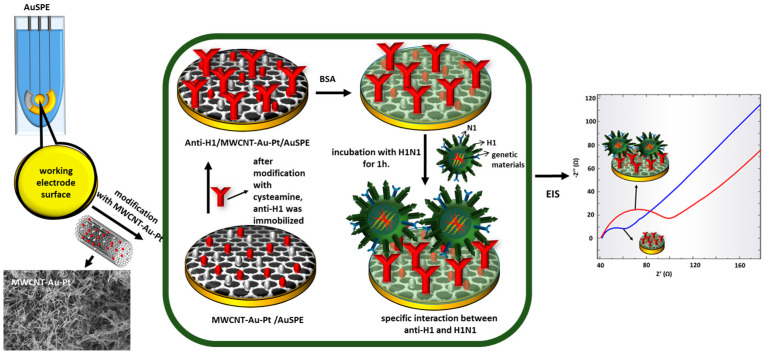
Schematic illustration of the fabrication of developed anti-H1 based MWCNT-Au-Pt hybrid nanomaterial-modified electrochemical influenza A biosensor.

In this study, AuSPE was used as a transducer. The first step involved the modification of AuSPE with MWCNT-Au-Pt hybrid nanomaterial. For this purpose, 10 µL of MWCNT-Au-Pt suspension (10 mg/mL in pure water) was dropped onto the working electrode surface and left until the suspension dehydrated at 25 °C for 15 min. Next, MWCNT-Au-Pt hybrid nanomaterial-modified electrode surface was treated with 10µL, 100 mM of cysteamine for 1 h for the formation of –NH_2_ functional groups on the surface. Then, 10 µL, 60 µg/mL anti-H3 antibodies in the presence of 10 µL, 5 mM of EDC, and 8 mM of NHS linker reagents mixture were immobilized on the electrode surface by waiting for 1 h in order to achieve a specific bioactive layer. After the antibody immobilization, 10 µL of 0.1% BSA was placed on the electrode surface as a blocking solution for the prevention of nonspecific bindings. After each step, the electrodes were washed with pH 7.4, 50 mM phosphate buffer. The fabricated anti-H1-based electrochemical immunosensors were prepared for single-use (Scheme 1). 

### 2.6. Electrochemical H1N1 Virus Detection Procedure

Different concentrations of 10 µL of H1N1 virus solutions were incubated with developed influenza A immunosensor at 25 °C for 1 h. For the investigation of interactions between H1N1 virus and anti-H1 antigen which was immobilized onto the working electrode, EIS measurements were carried out in 50 mM pH 7.4 phosphate buffer including 10 mM [Fe(CN)_6_]^4−^/[Fe(CN)_6_]^3−^ as a redox probe indicator in the frequency range of 0.1 Hz–10 kHz and with a potential of 0.10 V. Each experiment was carried out in triplicate, and the results were presented with error bars.

### 2.7. Sample Application

In total, 10 µL of 5 µg/mL H1N1 virus solution was put in synthetic saliva solution and incubated with developed anti-H1-based electrochemical influenza A immunosensor. A synthetic saliva solution was prepared by incorporating the reagents into pH 7.3 phosphate buffer including 0.05 mol/L NaHCO_3_, 8.55 × 10^−3^ mol/L NaCl and 2.68 × 10^−3^ mol/L KCl [39]. Next, electrochemical measurements were performed by using EIS in triplicate.

### 2.8. Selectivity Study

In order to appraise the selectivity of developed anti-H1-based electrochemical influenza A immunosensor, EIS measurements were performed to monitor the resistance changes on the electrode surface after incubating developed immunosensor with 10 µL, 5 µg/mL of the H1N1 and H3N2 influenza A viruses which were model and control viruses, respectively.

## 3. Results

### 3.1. Selection of Type of Hybrid Nanomaterial

**Figure 1:**
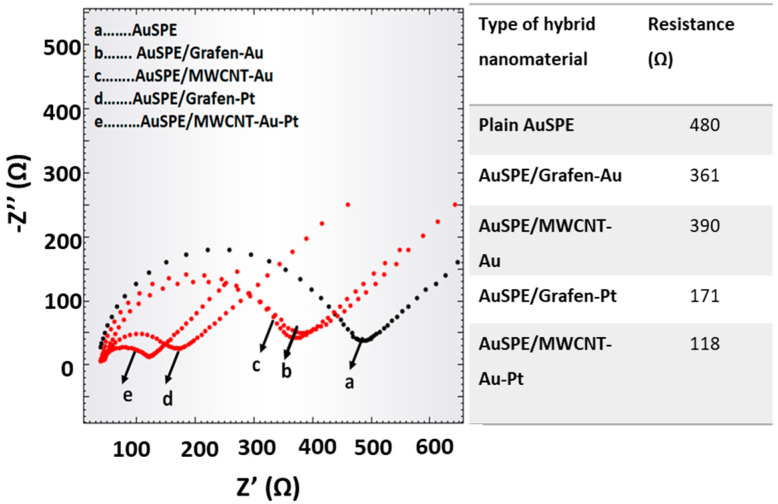
Nyquist plots and resistance values of plain AuSPE, AuSPE/graphene-Au, AuSPE/MWCNT-Au, AuSPE/graphene-Pt, and AuSPE/MWCNT-Au-Pt.

In order to select the most convenient hybrid nanomaterial, experiments by using plain AuSPE, AuSPE/Grafen-Au, AuSPE/MWCNT-Au, AuSPE/Grafen-Pt, and AuSPE/MWCNT-Au-Pt, were conducted via EIS in 50 mM pH 7.4 phosphate buffer including 10 mM [Fe(CN)_6_]^4−^/[Fe(CN)_6_]^3−^ as a redox probe in the frequency range of 0.1 Hz–10 kHz and with a potential of 0.10 V. Figure 1 shows the electrochemical performances of different types of hybrid nanomaterials. According to the resistance values obtained from the Nyquist plots, it is possible to say that MWCNT-Au-Pt/AuSPE has the lowest resistance value due to the high conductivity of the MWCNT-Au-Pt hybrid nanomaterial. For this reason, in order to increase the efficacy of the developed immunosensor, this hybrid nanomaterial was selected and used for further studies.

### 3.2. MWCNT-Au-Pt Hybrid Nanomaterial Characterization

**Figure 2:**
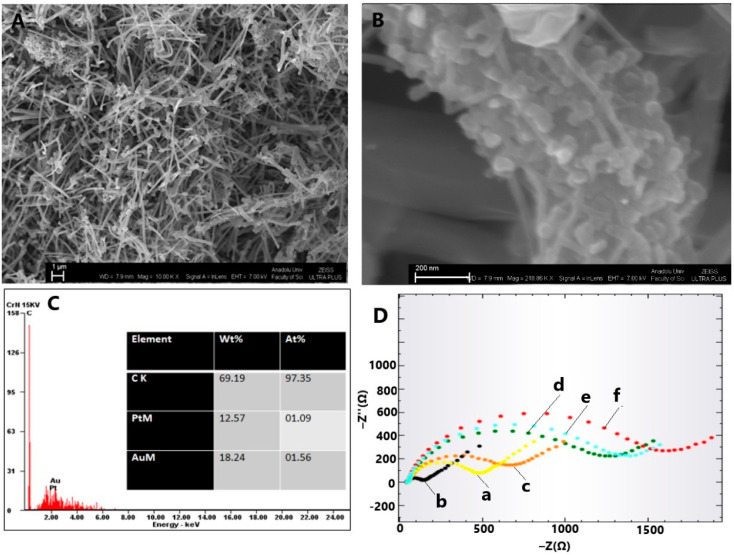
SEM images of the MWCNT-Au-Pt hybrid nanomaterial at (**A**) 1000× magnification, (**B**) 21,886× magnification, (**C**) EDX spectrum of the MWCNT-Au-Pt hybrid nanomaterial and (**D**) Nyquist plots of the developed immunosensor for electrochemical characterization (a. AuSPE, b. MWCNT-Au-Pt/AuSPE, c. Cysteamine/MWCNT-Au-Pt/AuSPE, d. Anti-H1/cysteamine/MWCNT-Au-Pt/AuSPE e. BSA/anti-H1/cysteamine/MWCNT-Au-Pt/AuSPE, f. H1N1 virus/BSA/anti-H1/cysteamine/MWCNT-Au-Pt/AuSPE).

SEM and EDX analyses were used to provide information regarding the morphological and structural composition of the synthesized MWCNT-Au-Pt hybrid nanomaterial. Figure 2A shows the typical CNT shape in the form of sticks with a thickness of 400 nm. The deposited AuNPs and PtNPs on the surface of CNT were observed as round-shaped and homogeneously dispersed bright dots in Figure 2B. From SEM images, it is possible to say that AuNPs and PtNPs were accumulated together on the CNT structure. Additionally, the sizes of these metal nanomaterials on the MWCNT were obtained in the range of 45 to 75 nm.

In Figure 2C, EDX results confirmed that Au and Pt nanoparticles were successfully deposited on MWCNT surface. The weight and atomic percentages of the elements in the prepared hybrid nanomaterial structure were determined as 69.19% C, 12.57% Pt, and 18.24% Au, and 97.35% C, 1.09% Pt, and 1.56% Au, respectively. 

### 3.3. Electrochemical Characterization of Developed Biosensor

EIS was used for the layer-by-layer electrochemical characterization of developed influenza A immunosensor. The Nyquist plots consist of two parts which are a semicircle and a straight line. Whereas the straight line demonstrates the Warburg impedance, which is due to diffusion at low frequencies, the semicircle diameter represents the charge transfer resistance on the electrode surface. As shown in Figure 2D, compared to the bare gold working electrode surface resistance, MWCNT-Au-Pt hybrid nanoparticle-modified AuSPE has a smaller semicircle signal due to the facile electron transfer process because of the conductivity that was provided by AuNP structure (a,b). Then, when anti-H1 was immobilized onto the electrode surface after modification with cysteamine (c), the semicircle diameter became bigger owing to harder electron transfer (d). At this stage, the electrode surface was saturated with BSA to prevent non-specific binding, and for this reason, the radius of the semicircle became bigger (e). Next, based on the specific binding of H1 proteins on H1N1 virus to anti-H1 antibodies on developed immunosensor surface, the blocking layer originating from the bound H1N1 virus was formed on the electrode surface. Therefore, as the electron transfer became harder because of that layer, the biggest semicircle was obtained (f). 

### 3.4. Experimental Parameters Optimization

**Figure 3:**
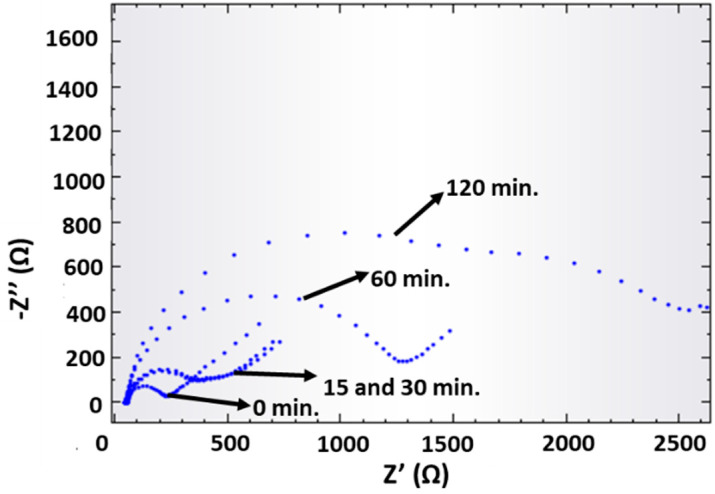
Nyquist plots that were obtained before (0 min) and after (15, 30, 60, 120 min) the interaction of MWCNT-Au-Pt/AuSPE with cysteamine.

Because of the presence of –SH and –NH_2_ functional groups in its structure, cysteamine plays a significant role in binding the biological materials onto the gold surface. Without any pre-thiolation, cysteamine can bind AuNPs from -SH ends and biological materials from -NH_2_ ends [40,41]. As mentioned in the experimental part, the electrode surface was activated with cysteamine so that the antibodies could bind effectively onto the electrode surface. For this reason, a series of experiments were carried out to determine the optimum time required for the modification of the electrode surface. According to Nyquist plots that were obtained before (0 min) and after (15, 30, 60, 120 min) the interaction with cysteamine (Figure 3), it was seen that the optimum modification time is 60 min. When the modification time was increased, the formation of a double semicircle curve was observed. This situation may be explained as the oxidation of -SH ends of the cysteamine into -SO_2_ and -SO_3_ in a long waiting time. In addition, there is a possibility that -COOH ends in the MWCNT-Au-Pt structure on the electrode surface may have an additional interaction with cysteamine [42,43].

**Figure 4:**
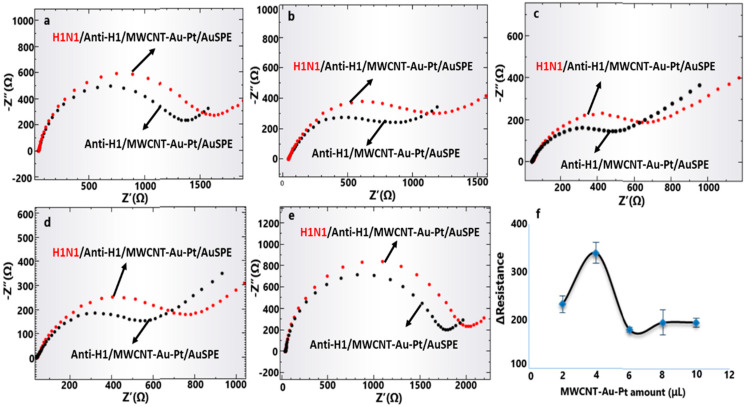
Nyquist plots for electrochemical influenza A immunosensor that contained different amounts of MWCNT-Au-Pt hybrid nanomaterials: (**a**) 2 µL, (**b**) 4 µL, (**c**) 6 µL, (**d**) 8 µL, (**e**) 10 µL (10 µL from 10 mg/mL suspension), and (**f**) change in the resistance values versus increasing the MWCNT-Au-Pt hybrid amount.

Moreover, MWCNT-Au-Pt hybrid nanomaterial amount in the developed immunosensor structure was also optimized. For this purpose, five electrochemical influenza A immunosensors that contain different amounts of MWCNT-Au-Pt hybrid nanomaterials (2, 4, 6, 8, and 10 µL from 10 mg/mL suspension) and 60 µg/mL of anti-H1 antibody were prepared, and then the responses against 7.5 µg/mL of H1N1 virus detection were investigated (Figure 4). As can be seen in Figure 4f, the best result was obtained with 4 µL of the MWCNT-Au-Pt hybrid nanomaterial-included immunosensor. Using more than 4 µL of MWCNT-Au-Pt hybrid nanomaterial in the immunosensor structure resulted in a decrease in the resistance value (Figure 4f). This may be related to the limited active surface area due to the excessive and irregular immobilization of MWCNT-Au-Pt hybrid nanomaterial on the electrode surface [44,45].

**Figure 5:**
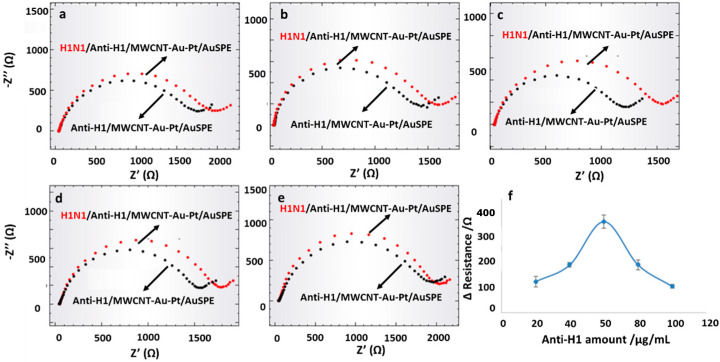
Nyquist plots for the electrochemical influenza A immunosensor that contained different amounts of anti-H1 antibody: (**a**) 20 µg/mL, (**b**) 40 µg/mL, (**c**) 60 µg/mL, (**d**) 80 µg/mL, (**e**) 100 µg/mL, and (**f**) change in the resistance values towards increasing concentrations of anti-H1 antibody.

The detection ability of the electrochemical biosensor depends on bioactive layer preparation procedure and interaction conditions between that layer and the biological analyte [43,46,47]. Therefore, in order to determine the electrode surface saturation concentration of anti-H1 antibody, EIS measurements were conducted with the developed immunosensor containing different concentrations of anti-H1 (20, 40, 60, 80, and 100 µg/mL) and 4 µL of MWCNT-Au-Pt hybrid nanomaterial after the interaction with 7.5 µg/mL of H1N1 virus (Figure 5). As can be seen from Figure 5f, the maximum resistance difference was obtained with 60 µg/mL anti-H1-included influenza A immunosensor. When more concentrated anti-H1 antibody was used in the developed immunosensor, the obtained resistance value decreased. This can be explained by the oversaturation of the bioactive layer when using anti-H1 antibodies more concentrated than 60 µg/mL [48]. 

### 3.5. Analytical Characteristics

**Figure 6:**
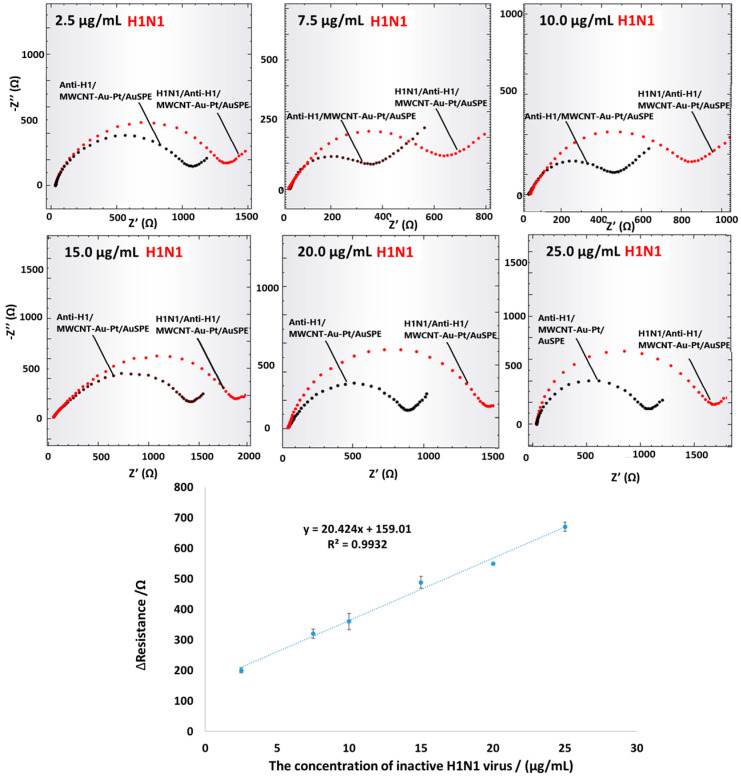
Nyquist plots and calibration graph of developed immunosensor. EIS measurements were performed in 50 mM pH 7.4 phosphate buffer including 10 mM [Fe(CN)_6_]^4−^/[Fe(CN)_6_]^3−^ as a redox probe indicator, in the frequency range of 0.1 Hz–10 kHz at a potential of 0.10 V.

After the experimental parameters were optimized, analytical characteristics of the developed system towards different concentrations (2.5, 7.5, 10, 15, 20, 25 µg/mL) of H1N1 were examined with EIS technique (Figure 6). According to the obtained calibration curve, as shown in Figure 6, the developed electrochemical influenza A immunosensor shows a linear response over H1N1 virus concentration range of 2.5–25.0 µg/mL (R^2^ = 0.99) with the linear regression equation of y = 20.424x + 159.01. In addition, limit of detection (LOD; 3 s/m; s is the standard deviation of blank solution for 3 points, m is the slope of the calibration plot) and limit of quantification (LOQ; 10 s/m) values were calculated as 3.54 µg/mL and 11.80 µg/mL, respectively. Relative standard deviation (RSD) value was found as 0.45% for 20 µg/mL concentration of the H1N1 virus (*n* = 3).

### 3.6. Selectivity Study

**Figure 7:**
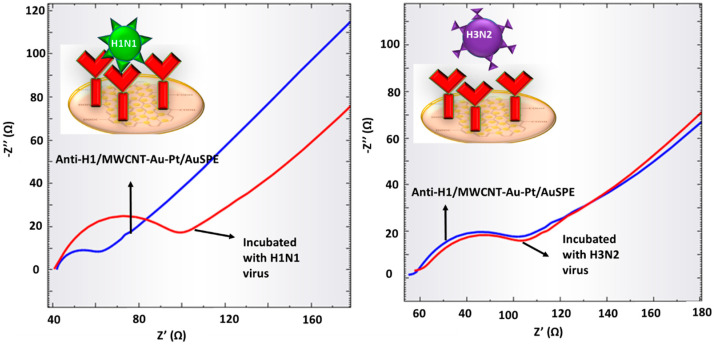
Obtained Nyquist plots after the incubation of H1N1 and H3N2 influenza A viruses with developed electrochemical anti-H1-based influenza A immunosensor.

Selectivity study was performed by using 10 µL of 5 µg/mL H1N1 and H3N2 viruses. When the developed MWCNT-Au-Pt hybrid nanomaterial-modified anti-H1 antibody-based electrochemical immunosensor for influenza A detection was incubated with H3N2 virus for 1 h, no change was observed in the resistance value, whereas a remarkable increase in the resistance value was determined after the interaction with H1N1 virus. The obtained results demonstrates specific binding of H1 protein on H1N1 virus to anti-H1 antibody. Moreover, as expected, since H3 proteins on H3N2 virus do not react with anti-H1 antibody, no response was observed for this type of virus (Figure 7).

### 3.7. Sample Application

The matrix effect was examined using synthetic saliva supplemented with H1N1 virus. For this purpose, EIS measurements were performed with developed influenza A immunosensor after the incubation with a synthetic saliva solution containing 5 µg/mL of H1N1 influenza A virus for 1 h. According to the obtained resistance values, recovery value was calculated as 99.18% ± 0.17.

Table 1 shows the comparison of developed influenza A immunosensor with recent similar studies. As can be seen from Table 1, we can say that this study is at a good level in terms of linear range and LOD values.

## 4. Conclusions

In conclusion, an MWCNT-Au-Pt hybrid nanomaterial-modified anti-H1-based electrochemical immunosensor was produced for influenza A detection. Throughout the entire study, inactive H1N1 influenza A virus was used as a model virus. This developed platform has a potential application to diagnose different types of influenza viruses that contain the H1 protein on their surfaces. Besides being selective, sensitive, and practical, due to SPE usage, very small volumes of samples were analyzed with the developed immunosensor. In addition, we believe that developed system is of great importance because it can be adapted to all kinds of influenza virus determinations for the future just by changing the antibody type.

## Figures and Tables

**Table 1:** Comparison of the developed anti-H1 immunosensor’s performance with similar influenza biosensors developed in recent years.

Transducer	Bioactive Surface	Analyte	Method	LOD	Linear Detection Range	Reference
Au/MHA	H7N1 specific antibody	H7N1 virus	EIS	5 µg/mL	5–15 µg/mL	[49]
Gold electrode/protein A	Monoclonal M1 antibody	Human influenza virus	QCM	10^3^ pfu/mL (10 µg/mL)	10^3^–10^4^ pfu/mLNp	[50]
Au/MUA/4-APBA	SA	HA	QCM	0.26 μg mL^−1^	0.01–0.16 mM	[51]
Au/MUA/4-APBA	SA	HA	SPR	0.72 μg mL^−1^	0.01–0.16 mM	[51]
AuSPE/graphene-gold hybrid nanoparticle	Fetuin A (a sialic acid source)	Neu	EIS	10^−8^ U mL^−1^ of Neu	10^−8^ U mL^−1^ and 10^−1^ U mL^−1^ of Neu (H9N2 virus)	[10]
Gold electrode/nanoporous gold film	H5N1 AIV ssDNA aptamer	H5N1 avian influenza virus	QCM	2^−4^ HAU/50 μL	2^−4^ to 2^4^ HAU/50 μL	[52]
Screen-printed IDAE (gold)	H5N1 specific aptamer	H5N1 virus	EIS	8 × 10^−4^ HAU in 200 μL sample	0.001–1 HAU in 200 μL sample	[53]
ITO/AuNPs	H7N9 monoclonal antibody	H7N9	ELISA	7.8 fg/mL	0.01–1.5 pg/mL	[54]
OECT	2,6-sialyllacose	Human influenza virus	Amperometric	0.025 HAU	0.03–1 HAU	[55]
Diamond electrode	polyclonal anti-M1 protein antibody	H1N1 and H3N2 virus	EIS	1 fg/mL virus	Up to 100 fg/mL virus	[56]
Gold surface	New generated H7 antibody	H9N7 virus	Intensity-modulated SPR	402 copies/mL	2.3 × 10^2^ to 2.3 × 10^5^ copies/mL	[57]
AuSPE/ MWCNT-Au-Pt	H1 antibody	H1N1 virus	EIS	3.54 µg/mL of H1N1	2.50–25.00 µg/mL of H1N1	This Study

OT + BDDT/NV/Ab: neutravidin-biotinylated antibody; MUA: 11-mercaptoundecanoic acid; 4-APBA: 4-Aminophenyl boronic acid; QCM: quartz crystal microbalance, SPR: surface plasmonurface plasmon resonance, HA: hemagglutinin, SA: sialic acid; IAV: influenza A virus, Neu: neuraminidase; HAU: hemagglutination units; ELISA: enzyme-linked immunoassay; OECT: organic electrochemical transistor; IDAE: interdigitated array gold electrode.

## Data Availability

Data will be made available on request.
